# Patterns and Factors Associated With Adherence to Lung Cancer Screening in Diverse Practice Settings

**DOI:** 10.1001/jamanetworkopen.2021.8559

**Published:** 2021-04-30

**Authors:** Lori C. Sakoda, M. Patricia Rivera, Jie Zhang, Pasangi Perera, Cecile A. Laurent, Danielle Durham, Roger Huamani Velasquez, Lindsay Lane, Adam Schwartz, Charles P. Quesenberry, George Minowada, Louise M. Henderson

**Affiliations:** 1 Division of Research, Kaiser Permanente Northern California, Oakland; 2 Department of Health System Science, Kaiser Permanente Bernard J. Tyson School of Medicine, Pasadena, California; 3 Division of Pulmonary and Critical Care Medicine, Department of Medicine, The University of North Carolina at Chapel Hill, Chapel Hill; 4 Lineberger Comprehensive Cancer Center, The University of North Carolina at Chapel Hill, Chapel Hill; 5 Department of Radiology, The University of North Carolina at Chapel Hill, Chapel Hill; 6 Department of Pulmonary Medicine, Kaiser Permanente Northern California, Vallejo; 7 Department of Epidemiology, The University of North Carolina at Chapel Hill, Chapel Hill

## Abstract

**Importance:**

For lung cancer screening to confer mortality benefit, adherence to annual screening with low-dose computed tomography scans is essential. Although the National Lung Screening Trial had an adherence rate of 95%, current data are limited on screening adherence across diverse practice settings in the United States.

**Objective:**

To evaluate patterns and factors associated with adherence to annual screening for lung cancer after negative results of a baseline examination, particularly in centralized vs decentralized screening programs.

**Design, Setting, and Participants:**

This observational cohort study was conducted at 5 academic and community-based sites in North Carolina and California among 2283 individuals screened for lung cancer between July 1, 2014, and March 31, 2018, who met US Preventive Services Task Force eligibility criteria, had negative results of a baseline screening examination (American College of Radiology Lung Imaging Reporting and Data System category 1 or 2), and were eligible to return for a screening examination in 12 months.

**Exposures:**

To identify factors associated with adherence, the association of adherence with selected baseline demographic and clinical characteristics, including type of screening program, was estimated using multivariable logistic regression. Screening program type was classified as centralized if individuals were referred through a lung cancer screening clinic or program and as decentralized if individuals had a direct clinician referral for the baseline low-dose computed tomography scan.

**Main Outcomes and Measures:**

Adherence to annual lung cancer screening, defined as a second low-dose computed tomography scan within 11 to 15 months after baseline screening.

**Results:**

Among the 2283 eligible individuals (1294 men [56.7%]; mean [SD] age, 64.9 [5.8] years; 1160 [50.8%] aged ≥65 years) who had negative screening results at baseline, overall adherence was 40.2% (n = 917), with higher adherence among those who underwent screening through centralized (46.0% [478 of 1039]) vs decentralized (35.3% [439 of 1244]) programs. The independent factor most strongly associated with adherence was type of screening program, with a 2.8-fold increased likelihood of adherence associated with centralized screening (adjusted odds ratio [aOR], 2.78; 95% CI, 1.99-3.88). Another associated factor was age (65-69 vs 55-59 years: aOR, 1.38; 95% CI, 1.07-1.77; 70-74 vs 55-59 years: aOR, 1.47; 95% CI, 1.10-1.96).

**Conclusions and Relevance:**

After negative results of a baseline examination, adherence to annual lung cancer screening was suboptimal, although adherence was higher among individuals who were screened through a centralized program. These results support the value of centralized screening programs and the need to further implement strategies that improve adherence to annual screening for lung cancer.

## Key Points

**Question:**

What are the patterns and factors associated with adherence to annual screening for lung cancer across diverse practice settings?

**Findings:**

In this cohort study, adherence to annual screening was suboptimal overall but was higher among individuals who were screened through centralized vs decentralized programs. The other significant factor associated with adherence was age.

**Meaning:**

This study suggests that centralized screening programs facilitate greater adherence to annual screening for lung cancer; further efforts to improve adherence need to be adopted to realize the mortality benefit associated with lung cancer screening.

## Teaser

This cohort study evaluates patterns and factors associated with adherence to annual screening for lung cancer after negative results of a baseline examination, particularly in centralized vs decentralized screening programs.

## Introduction

Landmark results from the National Lung Screening Trial (NLST) support recommendations for lung cancer screening (LCS) with low-dose computed tomography (LDCT) in the United States.^1,2,3^ The NLST demonstrated that annual screening of high-risk adults with LDCT (vs chest radiography) led to a 20% reduction in lung cancer mortality.^4^ The Dutch-Belgian NELSON (Nederlands-Leuvens Longkanker Screenings Onderzoek) trial subsequently showed a mortality benefit from LDCT screening,^5^ substantiating LCS as an effective strategy to reduce the high mortality rate of lung cancer worldwide. Since the publication of the 2013 United States Preventive Services Task Force (USPSTF) and Centers for Medicare & Medicaid Services recommendations, which endorse annual LDCT screening of adults aged 55 to 77 years or 80 years with a smoking history of 30 or more pack-years and less than 15 years since quitting, the uptake of LCS has been increasing nationally.^6,7,8,9^ However, for LCS to achieve similar long-term effectiveness in real-world practice, successful completion of multiple steps, including individuals returning for annual LDCT screening, is vital.^10^

In the NLST and NELSON trials, adherence to annual LDCT screening was more than 90%.^4,5^ Adherence below that observed in these trials may decrease the benefit to harm ratio of LCS, and current USPSTF recommendations are based on modeling studies that estimated the mortality benefit associated with various LDCT screening scenarios assuming 100% adherence.^11^ In practice, reported adherence has been variable and lower than 100%, ranging from 18% to 86%.^12,13,14,15,16,17,18^ This variation is likely explained by differences in institutional practices around the implementation of LCS programs, the populations screened, and the applied definition of screening adherence. In particular, adherence to annual LDCT screening is likely higher in centralized vs decentralized screening programs. Unlike decentralized programs, centralized programs, or “hub and spoke” models, generally have a dedicated team that includes clinicians and nurse coordinators and the infrastructure to manage and support the screening process, which may include tracking and notifying individuals that they are due for screening and facilitating the actions needed to return for screening examinations. To our knowledge, the potential difference in adherence associated with program type has not been well examined because most studies examining factors associated with adherence have been conducted in single institutions, where there is often no variability in program type. Adherence to annual LDCT screening should also be assessed strictly after negative results of a screening examination, as more frequent follow-up is recommended after positive results of a screening examination, and in those who remain eligible for annual screening. At present, data on adherence remain limited, especially in community practice settings, yet they are fundamental in guiding interventions and policy decisions to optimize LCS effectiveness.

To address this knowledge gap, we report on the patterns and factors associated with adherence to LCS after negative results from a baseline LDCT examination across diverse practice settings in the US. Given evidence suggesting that support of a program coordinator and patient reminders promote better adherence in academic settings,^13,15^ we focused on examining whether adherence was higher among individuals initially screened through centralized vs decentralized LCS programs.

## Methods

### Study Settings

Our study included 5 screening sites: 4 academic or community-based sites in central and eastern North Carolina and a large community-based integrated health system in northern California. At the sites in North Carolina, eligible individuals are referred for LCS in several ways. Individuals may be referred to LCS clinics or programs by their primary care physician (PCP), by subspecialty or other health professionals (eg, pulmonologists), or by self-referral. In these scenarios, individuals engage in shared decision-making, and if they choose to undergo the LDCT examination, they are followed up directly by the LCS clinic or program clinicians thereafter. Alternatively, PCPs and subspecialty clinicians may directly refer individuals for LDCT examinations, bypassing the LCS clinics or programs. In this scenario, individuals undergo shared decision-making with their PCP or subspecialist, and if they choose to undergo the LDCT examination, they are followed up by their respective clinicians. For the health system in California, screening workflows evolved and changed over time. When LCS was first implemented, PCPs directly referred individuals for baseline LDCT examinations.^19^ A regional LCS program integrating clinical navigation was later rolled out, whereby PCPs hand off potentially eligible individuals to designated clinician specialists who are responsible for verifying screening eligibility, providing education, conducting shared decision-making, and ordering baseline LDCT examinations.^20^ For comparability and consistency across study sites, we considered receipt of a direct clinician referral for the baseline LDCT examination as being screened through a decentralized program and receipt of a referral through an LCS clinic or program for the baseline LDCT examination as being screened through a centralized program. Individuals at the health system in California were therefore classified as being screened through either a centralized or decentralized program, based on whether they were initially screened or not screened through the regional LCS program. Our definition of program type follows that of others.^21^ The institutional review boards of the University of North Carolina at Chapel Hill and Kaiser Permanente Northern California approved this study with a waiver of informed consent, because seeking informed consent from all individuals included in the study was unfeasible and the risk to participants was minimal. This study followed the Strengthening the Reporting of Observational Studies in Epidemiology (STROBE) reporting guideline for cohort studies.

### Study Design and Population

Our study population was drawn from individuals who underwent a baseline LDCT screening examination during the period from July 1, 2014, to March 31, 2018. Screening eligibility was defined following the 2013 USPSTF LCS recommendations,^1^ specifically: adults aged 55 to 80 years with a 30 or more pack-year smoking history who currently smoke or had quit within the past 15 years. Our study inclusion period was limited to ensure that all individuals had at least 15 months of follow-up after their baseline screening examination (ie, follow-up through June 30, 2019). The American College of Radiology Lung Imaging Reporting and Data System (Lung-RADS) version 1.0 was used to standardize reporting and management of LDCT screening results at all study sites.^22^ The Lung-RADS classifies LDCT screening results into 4 major assessment categories, with LDCT screening in 12 months recommended for negative examination results (Lung-RADS category 1 or 2) and either immediate diagnostic evaluation or follow-up LDCT screening in 3 or 6 months recommended for positive examination results (Lung-RADS category 3, 4A, 4B, or 4X).

We strictly examined individuals who had negative results of baseline LDCT examination (ie, Lung-RADS category 1 or 2), with a recommendation to return for LDCT screening in 12 months. Accordingly, we excluded those who were first screened at 80 years of age or whose radiology report did not specify a Lung-RADS classification. Adherence was defined as LDCT screening within 11 to 15 months after the date of the negative baseline LDCT examination results. We therefore also excluded those who died within 15 months after their baseline LDCT examination. Individuals screened at the health system in California were further required to have at least 15 months of continuous health plan membership after their baseline LDCT examination to ensure that lack of adherence was not due to health plan disenrollment.

### Data Sources and Elements

We established guidelines to acquire the same data elements from electronic health record databases across sites, which we used to systematically identify and characterize eligible individuals. Data ascertained as potential factors associated with adherence to screening measured at the baseline LDCT examination included age, sex, race/ethnicity, place of residence (urban or rural), smoking status (current, former, or unspecified [ie, only documented as current or former]), history of chronic obstructive pulmonary disease (COPD), history of invasive cancer, and type of screening program (centralized or decentralized). We considered only factors that we could consistently capture using discrete electronic health record data from all sites and that we hypothesized could be associated with adherence. Classification for place of residence was evaluated by linking residential zip code to the 2013 US Department of Agriculture rural-urban continuum codes.^23^ Documented *International Classification of Diseases, Ninth Revision* (*ICD-9*) and *International Statistical Classification of Diseases and Related Health Problems, Tenth Revision* (*ICD-10*) codes were used to evaluate history of COPD (*ICD-9* codes: 490.*, 491.*, 492.*, and 496.*; *ICD-10* codes: J40.*, J41.*, J43.*, and J44.*) and invasive cancer (*ICD-9* codes: V10.*; *ICD-10* code: Z85.*). All of the individuals who were included met screening eligibility on smoking history; however, we could not examine pack-years and time since quitting smoking because these data were captured only as either less than 30 or 30 or more pack-years and 15 or less or more than 15 years since quitting smoking from 1 study site.

### Statistical Analyses

To characterize patterns of screening adherence, we calculated the duration (in months) from baseline to next LDCT screening examination and the proportion of participants who were adherent, overall and separately by program type. We also calculated the proportion of participants who were adherent within categories of selected baseline characteristics.

To identify factors associated with adherence to screening, we estimated odds ratios (ORs) and 95% CIs for the association between baseline characteristics and screening adherence, overall and stratified by program type, using logistic regression. Regression models were constructed first for each characteristic individually and then for all characteristics combined, including screening site to account for variation across sites. Because all characteristics were selected as potential factors associated with adherence a priori, we included all characteristics in our final model. All statistical tests were 2-sided, with a type I error probability of 5%.

To enable direct comparisons with the NLST population, the analyses were repeated, limited to individuals aged 55 to 74 years at baseline.^4^ Analyses were conducted using SAS, version 9.4 (SAS Institute Inc).

## Results

### Baseline Characteristics

Our study included 2283 individuals screened for lung cancer with a recommendation to return for annual LDCT screening (Table 1). Participants’ mean (SD) age was 64.9 (5.8) years at their baseline LDCT examination. A larger proportion were male (1294 [56.7%]), White (1631 [78.5%]), currently smoking (1040 [55.4%]), and urban area residents (1930 [84.5%]). A total of 273 participants (12.0%) had a documented history of cancer, and 787 (34.6%) had a documented history of COPD. Although slightly more individuals were screened through a decentralized (1244 [54.5%]) vs centralized (1039 [45.5%]) program, their distributions were comparable on age and sex. Those screened through a decentralized program were more commonly individuals who were non-White (300 of 1230 [24.4%] vs 147 of 848 [17.3%]), previously smoked (407 of 870 [46.8%] vs 430 of 1007 [42.7%]), and resided in urban areas (1093 of 1244 [87.9%] vs 837 of 1039 [80.6%]). Also, a larger proportion of individuals screened through a decentralized program had a history of COPD (568 of 1243 [46.0%] vs 219 of 1039 [21.1%]), yet a smaller proportion had a history of cancer (106 of 1234 [8.6%] vs 167 of 1039 [16.1%]).

**Table 1.** Baseline Characteristics, Overall and by Type of Screening Program

Characteristic	Individuals, No. (%)		
	Overall (N = 2283)	Decentralized (n = 1244)	Centralized (n = 1039)
Age, y			
55-59	509 (22.3)	273 (21.9)	236 (22.7)
60-64	614 (26.9)	335 (26.9)	279 (26.9)
65-69	653 (28.6)	356 (28.6)	297 (28.6)
70-74	386 (16.9)	212 (17.0)	174 (16.7)
75-79	121 (5.3)	68 (5.5)	53 (5.1)
Age, mean (SD), y	64.9 (5.8)	64.9 (5.9)	64.8 (5.8)
Sex			
Male	1294 (56.7)	711 (57.2)	583 (56.1)
Female	989 (43.3)	533 (42.8)	456 (43.9)
Race/ethnicity^a^			
White	1631 (78.5)	930 (75.6)	701 (82.7)
Non-White	447 (21.5)	300 (24.4)	147 (17.3)
Black or African American	247 (11.9)	170 (13.8)	77 (9.1)
Asian	75 (3.6)	45 (3.7)	30 (3.5)
Hispanic	73 (3.5)	56 (4.6)	17 (2.0)
Other^b^	52 (2.5)	29 (2.4)	23 (2.7)
Unknown, No.	205	14	191
Smoking status^a^			
Current	1040 (55.4)	463 (53.2)	577 (57.3)
Former	837 (44.6)	407 (46.8)	430 (42.7)
Unspecified: current or former	406	374	32
Place of residence			
Urban	1930 (84.5)	1093 (87.9)	837 (80.6)
Rural	353 (15.5)	151 (12.1)	202 (19.4)
History of cancer^a^			
Yes	273 (12.0)	106 (8.6)	167 (16.1)
No	2000 (88.0)	1128 (91.4)	872 (83.9)
Unknown, No.	10	10	0
History of COPD^a^			
Yes	787 (34.6)	568 (46.0)	219 (21.1)
No	1486 (65.4)	666 (54.0)	820 (78.9)
Unknown, No.	10	10	0

Abbreviation: COPD, chronic obstructive pulmonary disease.

^a^ Percentages were calculated excluding individuals who were classified as unknown or unspecified.

^b^ Other includes American Indian or Alaska Native, Native Hawaiian or Pacific Islander, multiple race/ethnicity, or any race/ethnicity not included in the other categories.

### Patterns of Adherence to Screening

**Figure 1.**
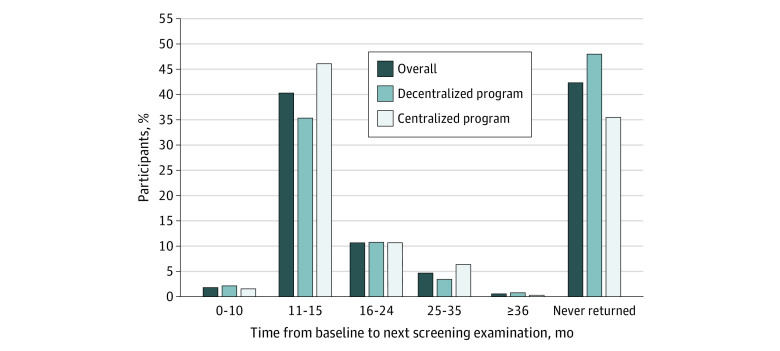
Percentage Distribution of Time from Baseline to Next Low-Dose Computed Tomography Screening Examination, Overall and by Type of Screening Program

Overall, 1319 individuals (57.8%) returned for LDCT screening at any time after their baseline screening examination, and 917 returned within 11 to 15 months, resulting in 40.2% adherence (Figure 1). The median time between the baseline LDCT screening examination and the second LDCT screening examination was 13.1 months (interquartile range, 12.1-16.4 months). When stratified by program type, individuals screened through a centralized program were proportionally more likely than those screened through a decentralized program to be adherent (46.0% [478 of 1039] vs 35.3% [439 of 1244]), and, in general, they were more likely to return for LDCT screening (64.6% [671 of 1039] vs 52.1% [648 of 1244]).

Greater adherence to screening was also evident in other subgroups, specifically individuals who were aged 65 to 74 years, were White, and previously smoked and had a history of invasive cancer or COPD (Table 2). When further stratified by program type, patterns of adherence were slightly different by sex, race/ethnicity, and history of cancer. Adherence was lower among men (vs women) and non-White (vs White) individuals screened through a decentralized program (230 of 711 [32.3%] men vs 209 of 533 [39.2%] women; 90 of 300 [30.0%] non-White individuals vs 344 of 930 [37.0%] White individuals) compared with those screened through a centralized program (282 of 583 [48.4%] men vs 196 of 456 [43.0%] women; 77 of 147 [52.4%] non-White individuals vs 390 of 701 [55.6%] White individuals). Among non-White individuals, adherence was the poorest among Asian individuals. In a subset analysis of the 70 Asian individuals with available data on language preference, adherence was 12% (3 of 25) for those having a preferred language other than English vs 31% (14 of 45) for those whose preferred language was English. Adherence was higher only among individuals with (vs without) a history of cancer who were screened through a centralized program. Patterns of adherence were similar when restricting to individuals aged 55 to 74 years at baseline (NLST-eligible participants; eTable 1 in the Supplement), despite adherence being the lowest (33.1% [40 of 121]) among those aged 75 to 79 years.

**Table 2.** Screening Adherence, Overall and by Subgroups

Characteristic	Individuals, No./total No. (%)		
	Overall (N = 2283)	Decentralized (n = 1244)	Centralized (n = 1039)
Overall adherence	917 (40.2)	439 (35.3)	478 (46.0)
Age, y			
55-59	180/509 (35.4)	81/273 (29.7)	99/236 (41.9)
60-64	244/614 (39.7)	112/335 (33.4)	132/279 (47.3)
65-69	275/653 (42.1)	137/356 (38.5)	138/297 (46.5)
70-74	178/386 (46.1)	89/212 (42.0)	89/174 (51.1)
75-79	40/121 (33.1)	20/68 (29.4)	20/53 (37.7)
Sex			
Male	512/1294 (39.6)	230/711 (32.3)	282/583 (48.4)
Female	405/989 (41.0)	209/533 (39.2)	196/456 (43.0)
Race/ethnicity			
White	734/1631 (45.0)	344/930 (37.0)	390/701 (55.6)
Non-White	167/447 (37.4)	90/300 (30.0)	77/147 (52.4)
Black or African American	92/247 (37.2)	52/170 (30.6)	40/77 (51.9)
Asian	19/75 (25.3)	9/45 (20.0)	10/30 (33.3)
Hispanic	29/73 (39.7)	19/56 (33.9)	10/17 (58.8)
Other^a^	27/52 (51.9)	10/29 (34.5)	17/23 (73.9)
Unknown	16/205 (7.8)	5/14 (35.7)	11/191 (5.8)
Smoking status			
Current	401/1040 (38.6)	154/463 (33.3)	247/577 (42.8)
Former	376/837 (44.9)	160/407 (39.3)	216/430 (50.2)
Unspecified: current or former	140/406 (34.5)	125/374 (33.4)	15/32 (46.9)
Place of residence			
Urban	770/1930 (39.9)	380/1093 (34.8)	390/837 (46.6)
Rural	147/353 (41.6)	59/151 (39.1)	88/202 (43.6)
History of cancer			
Yes	119/273 (43.6)	36/106 (34.0)	83/167 (49.7)
No	798/2000 (39.9)	403/1128 (35.7)	395/872 (45.3)
History of COPD			
Yes	348/787 (44.2)	230/568 (40.5)	118/219 (53.9)
No	569/1486 (38.3)	209/666 (31.4)	360/820 (43.9)

Abbreviation: COPD, chronic obstructive pulmonary disease.

^a^ Other includes American Indian or Alaska Native, Native Hawaiian or Pacific Islander, multiple race/ethnicity, or any race/ethnicity not included in the other categories.

### Factors Associated With Adherence to Screening

**Figure 2.**
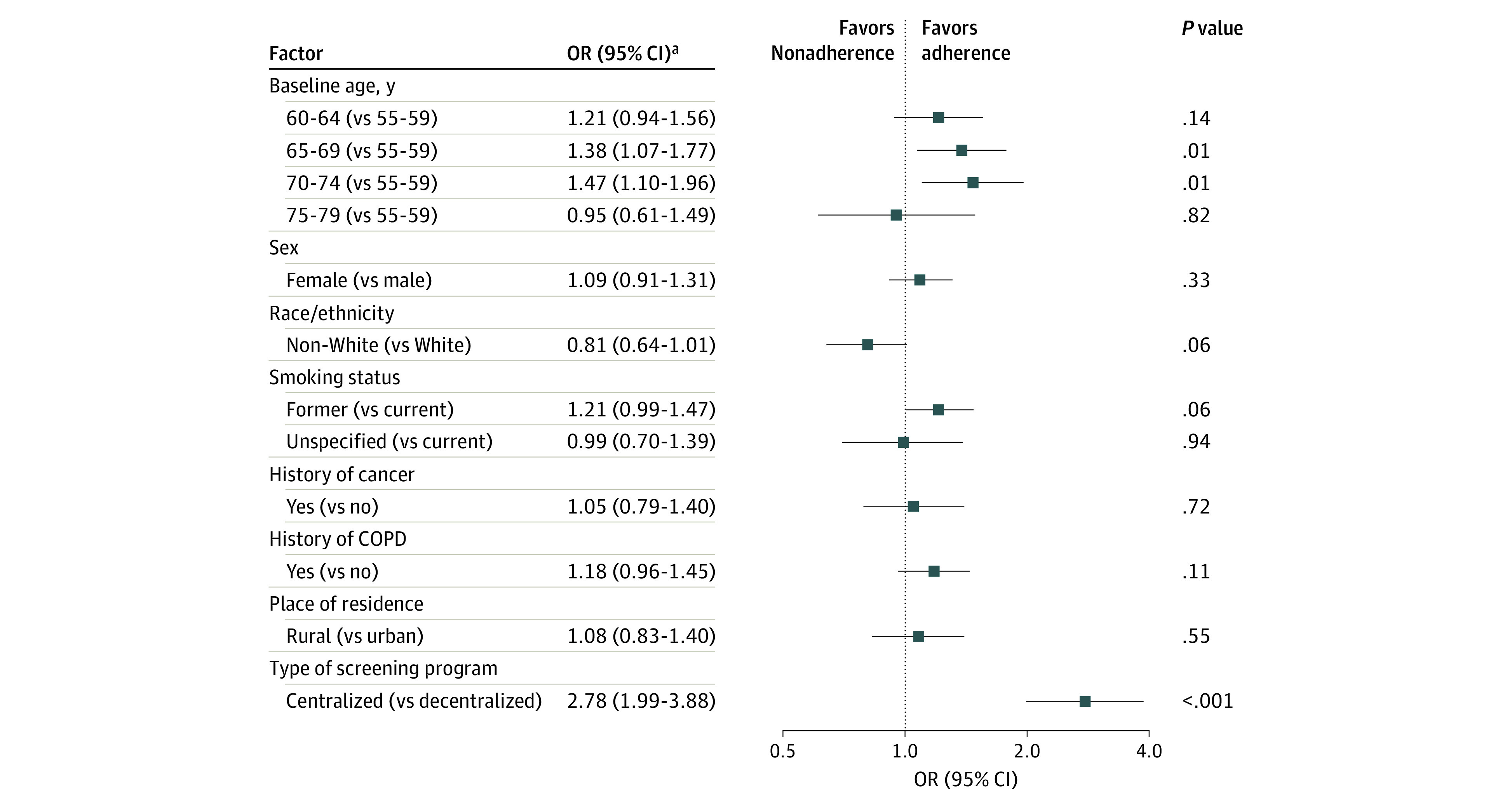
Factors Associated With Adherence After Negative Results of Baseline Low-Dose Computed Tomography Examination COPD indicates chronic obstructive pulmonary disease; and OR, odds ratio. ^a^Associations for each factor adjusted for all other factors shown and for screening site.

In our multivariable regression analyses (Figure 2), the strongest independent factor associated with adherence was program type, with individuals screened in a centralized program having more than a 2-fold higher likelihood of adherence compared with those screened in a decentralized program (adjusted OR [aOR], 2.78; 95% CI, 1.99-3.88). Higher adherence was also associated with baseline age (65-69 vs 55-59 years: aOR, 1.38; 95% CI, 1.07-1.77; 70-74 vs 55-59 years: aOR, 1.47; 95% CI, 1.10-1.96). There was no statistically significant association between adherence and race/ethnicity (aOR for non-White vs White, 0.81; 95% CI, 0.64-1.01), former smoking status (aOR, 1.21; 95% CI, 0.99-1.47), or history of COPD (aOR, 1.18; 95% CI, 0.96-1.45).

Higher adherence was similarly associated with baseline ages of 65 to 74 years, irrespective of program type (eTable 2 in the Supplement). However, for those screened through a decentralized program, female sex (aOR, 1.30; 95% CI, 1.02-1.65) and a history of COPD (aOR, 1.34; 95% CI, 1.05-1.71) were further associated with higher adherence. Race/ethnicity was not associated with adherence (aOR for non-White vs White, 0.79; 95% CI, 0.59-1.06). For those screened through a centralized program, no clear associations of sex, race/ethnicity, and a history of COPD with adherence were observed (eTable 2 in the Supplement). Results were again similar when restricting the sample to individuals aged 55 to 74 years at baseline (eTable 3 in the Supplement).

## Discussion

To our knowledge, this is the first examination of early patterns and factors associated with adherence to screening after negative results of a baseline LDCT examination comparing centralized vs decentralized screening programs in the US. Adherence was low overall but higher among individuals screened through a centralized program. Adherence was also higher among other subgroups, including those aged 65 to 74 years and those who previously smoked. Findings were materially unchanged when limiting analyses to NLST-eligible individuals.

Our data come from academic and community-based sites over a 45-month inclusion period, starting 6 months after the announcement of the first-ever USPSTF recommendation for annual LCS with LDCT. As a time of early adoption of LCS in clinical practice, it is not entirely surprising to find suboptimal adherence to recommended follow-up, especially after negative examination results. Several single-institution studies have found comparably low estimates of adherence after initial negative examination results, ranging from 18% to 51%.^13,14,17^ This seemingly wide variation is explained in part by the use of different definitions of adherence across studies, with the lowest estimate based on a second screening within 11 to 13 months and the highest estimate based on a second screening within 18 months.^13,14^ Nevertheless, others have reported higher estimates of adherence, up to 82%, when defining adherence as return for LDCT screening within 15 months.^12,15,16^ A common feature of these studies showing higher adherence is a centralized program with a dedicated program coordinator to support tracking and follow-up of screened individuals. At an academic medical center, adherence increased from 22% to 66% after hiring of a full-time LCS program coordinator.^15^ To date, the highest estimate of adherence (82%) has been documented by the Veterans Administration Lung Cancer Screening Demonstration Project, although even with its centralized program design, adherence varied considerably across sites.^16^

Our results further corroborate that individuals are more likely to be adherent to LCS when screened through centralized vs decentralized programs. In fact, the type of screening program emerged as the independent factor most strongly associated with adherence. Some studies have likewise found that individuals who are older (ie, 65-73 years) and who previously smoked are more likely to be adherent.^12,15^ In other studies, however, these and other demographic factors, including sex, race/ethnicity, and insurance status, have not been associated with adherence.^13,16,17^ Older individuals may be more adherent because they have better health care coverage (from Medicare) and more leisure time (from retirement), although our data also indicate that the oldest individuals (>74 years) may be the least adherent. In the context of guideline-concordant screening for breast, prostate, and colorectal cancers, individuals who currently smoke are reportedly less likely to be adherent to cancer screening regimens compared with those who have previously smoked or never smoked.^24^ The lower adherence among those who smoke reinforces the importance of promoting tobacco control for LCS to be effective in reducing lung cancer mortality. Findings of poorer adherence among younger individuals are especially noteworthy because the USPSTF recently issued recommendations to lower the age range for screening eligibility to 50 years.^25^

Our data raise some concern that individuals from racial/ethnic minority groups may have lower rates of adherence to LCS, particularly when they are screened through a decentralized program. Among minority groups, the lowest adherence was noted for Asian individuals. This disparity most likely arose from barriers in communication between patients and clinicians because adherence was substantially lower for those having a preferred language other than English (12% vs 31% for those whose preferred language was English). In a recent study of 201 Black individuals and 276 White individuals screened through a centralized program at an urban, academic medical center, Black race was the factor associated most strongly with decreased adherence to annual screening.^14^ Collectively, these findings affirm the importance of developing culturally sensitive shared decision-making aids and educational materials in other primary languages and at appropriate literacy levels to mitigate inequities in LCS.^26^

The mortality benefit associated with LCS requires high adherence to recommended follow-up care, including annual LDCT examinations. In the NLST intervention group, nearly 60% of the lung cancers were detected after the second and third rounds of LDCT screening.^4^ With microsimulation modeling, Han and colleagues found that when reducing LCS adherence from 100% to 39%, the estimated number of lung cancer deaths averted per 100 000 persons decreased from 501 to 230, a greater than 50% reduction in the benefit.^10^ Given the importance of high adherence to follow-up LCS, further work is needed to evaluate multilevel barriers to screening adherence, as well as to develop and implement effective interventions to overcome them.

### Limitations

This study has some limitations. First, our sample is not nationally representative. Selecting sites in California and North Carolina, however, enhanced the size and racial/ethnic diversity of individuals studied and enabled us to assess adherence by program type. Second, we likely underestimated adherence because we could identify and exclude only individuals screened in California who were lost to follow-up within 15 months owing to disenrollment; data were unavailable to similarly exclude individuals screened in North Carolina. Third, using electronic health record data precluded investigating additional factors that may be associated with adherence, including educational level, household income, employment status, and family history of lung cancer, and using clinical diagnosis codes to evaluate history of cancer and COPD likely resulted in some misclassification. Last, we examined adherence to only 1 round of screening after negative results of the baseline examination. As LCS programs mature and participation increases, trends in adherence to annual screening should be monitored for a longer duration.

## Conclusions

We found that adherence to annual LCS was suboptimal after negative results of a baseline LDCT examination. Higher adherence was associated with screening through a centralized program, supporting the investment and value in hiring dedicated program coordinators and implementing effective tracking systems. Our findings also reinforce the need for evidence-based practices and interventions to effectively and equitably support screening adherence.

## Notes

Supplement. **eTable 1.** Screening Adherence, Overall and by Subgroups, in NLST-Eligible Individuals **eTable 2.** Factors Associated With Screening Adherence After a Negative Baseline LDCT Examination for Decentralized and Centralized Screening Programs **eTable 3.** Factors Associated With Screening Adherence After a Negative Baseline LDCT Examination in NLST-Eligible Individuals
