# Supplementary material for: Patterns and Factors Associated With Adherence to Lung Cancer Screening in Diverse Practice Settings
Source: JAMA Netw Open. 2021 Apr 30;4(4):e218559. doi: 10.1001/jamanetworkopen.2021.8559 (PMC8087957; doi:10.1001/jamanetworkopen.2021.8559)
Supplement: Supplement. — eTable 1. Screening Adherence, Overall and by Subgroups, in NLST-Eligible Individuals eTable 2. Factors Associated With Screening Adherence After a Negative Baseline LDCT Examination for Decentralized and Centralized Screening Programs eTable 3. Factors Associated With Screening Adherence After a Negative Baseline LDCT Examination in NLST-Eligible Individuals [file jamanetwopen-e218559-s001.pdf]

Supplementary Online Content

Sakoda LC, Rivera MP, Zhang J, et al. Patterns and factors associated with adherence to lung cancer screening in diverse practice settings. *JAMA Netw Open.* 2021;4(4):e218559. doi:10.1001/jamanetworkopen.2021.8559

eTable 1. Screening Adherence, Overall and by Subgroups, in NLST-Eligible Individuals
eTable 2. Factors Associated With Screening Adherence After a Negative Baseline LDCT Examination for Decentralized and Centralized Screening Programs
eTable 3. Factors Associated With Screening Adherence After a Negative Baseline LDCT Examination in NLST-Eligible Individuals

This supplementary material has been provided by the authors to give readers additional information about their work.

© 2021 Sakoda LC et al. *JAMA Network Open.*

**eTable 1.** Screening Adherence, Overall and by Subgroups, in NLST-Eligible Individuals						
	**Overall (n=2,162)**		**Decentralized (n=1,176)**		**Centralized (n=986)**	
**Overall, No. (%)**	877	40.6	419	35.6	458	46.5
**Age**						
55-59	180	35.4	81	29.7	99	41.9
60-64	244	39.7	112	33.4	132	47.3
65-69	275	42.1	137	38.5	138	46.5
70-74	178	46.1	89	42.0	89	51.1
**Sex**						
Male	491	39.9	222	32.9	269	48.5
Female	386	41.4	197	39.3	189	43.9
**Race/Ethnicity**						
White	703	45.1	326	37.0	377	55.7
Non-White	158	38.2	90	32.1	77	57.5
Black/African American	85	37.9	50	32.5	35	50.0
Asian	17	23.6	9	20.0	8	29.6
Hispanic	29	42.0	19	35.2	10	66.7
Other	27	55.1	10	37.0	17	77.3
Unknown	16	8.5	5	35.7	11	6.3
**Smoking Status**						
Current	390	39.0	149	33.5	241	43.4
Former	349	44.7	147	38.7	202	50.4
Unspecified **-** Current or Former	138	36.2	123	35.0	15	50.0
**Place of Residence**						
Urban	739	40.4	366	35.4	373	47.0
Rural	138	41.2	53	37.3	85	44.0
**History of Invasive Cancer**						
Yes	112	46.7	34	37.8	78	52.0
No	765	40.0	385	35.8	380	45.5
**History of COPD**						
Yes	333	44.9	218	41.1	115	54.8
No	544	38.6	201	31.7	343	44.2

© 2021 Sakoda LC et al. *JAMA Network Open.*

eTable 2. Factors Associated With Screening Adherence After a Negative Baseline LDCT Examination for Decentralized and Centralized Screening Programs

	**Decentralized**		**Centralized**	
	**OR[a]**	**95% CI**	**OR[a]**	**95% CI**
**Age**				
55-59	1.00	reference	1.00	Reference
60-64	1.14	(0.81, 1.63)	1.31	(0.89, 1.92)
65-69	1.42	(1.01, 2.01)	1.38	(0.95, 2.01)
70-74	1.53	(1.04, 2.27)	1.45	(0.93, 2.25)
75-79	0.89	(0.49, 1.63)	1.07	(0.53, 2.15)
**Sex**				
Male	1.00	reference	1.00	Reference
Female	1.30	(1.02, 1.65)	0.91	(0.69, 1.20)
**Race/Ethnicity**				
White	1.00	reference	1.00	Reference
Non-White	0.79	(0.59, 1.06)	0.86	(0.59, 1.24)
**Smoking Status**				
Current	1.00	reference	1.00	Reference
Former	1.16	(0.87, 1.55)	1.24	(0.94, 1.65)
Unspecified - Current or Former	1.08	(0.73, 1.60)	0.62	(0.29, 1.32)
**History of Cancer**				
No	1.00	reference	1.00	Reference
Yes	0.92	(0.59, 1.44)	1.20	(0.82, 1.77)
**History of COPD**				
No	1.00	reference	1.00	Reference
Yes	1.34	(1.05, 1.71)	0.83	(0.57, 1.22)
**Place of Residence**				
Urban	1.00	reference	1.00	Reference
Rural	1.11	(0.76, 1.62)	1.06	(0.73, 1.52)

[a] Estimates for each factor adjusted for the other factors shown and screening site

© 2021 Sakoda LC et al. *JAMA Network Open.*

eTable 3. Factors Associated With Screening Adherence After a Negative Baseline LDCT Examination in NLST-Eligible Individuals

	Overall		Decentralized		Centralized	
	**OR[a]**	**95% CI**	**OR[a]**	**95% CI**	**OR[a]**	**95% CI**
**Age**						
55-59	1.00	reference	1.00	reference	1.00	reference
60-64	1.20	(0.93, 1.55)	1.14	(0.80, 1.62)	1.30	(0.88, 1.90)
65-69	1.36	(1.06, 1.75)	1.41	(1.00, 1.99)	1.37	(0.94, 1.99)
70-74	1.44	(1.08, 1.93)	1.52	(1.02, 2.24)	1.41	(0.91, 2.20)
**Sex**						
Male	1.00	reference	1.00	reference	1.00	reference
Female	1.09	(0.90, 1.31)	1.27	(0.99, 1.63)	0.93	(0.70, 1.22)
**Race/Ethnicity**						
White	1.00	reference	1.00	reference	1.00	reference
Non-White	0.83	(0.66, 1.05)	0.84	(0.62, 1.13)	0.84	(0.57, 1.24)
**Smoking Status**						
Current	1.00	reference	1.00	reference	1.00	reference
Former	1.16	(0.94, 1.42)	1.11	(0.83, 1.49)	1.19	(0.89, 1.59)
Unspecified - Current or Former	1.03	(0.72, 1.46)	1.10	(0.74, 1.65)	0.67	(0.31, 1.46)
**History of Invasive Cancer**						
No	1.00	reference	1.00	Reference	1.00	reference
Yes	1.16	(0.86, 1.56)	1.03	(0.64, 1.65)	1.30	(0.87, 1.95)
**History of COPD**						
No	1.00	reference	1.00	reference	1.00	reference
Yes	1.21	(0.98, 1.49)	1.37	(1.06, 1.75)	0.86	(0.58, 1.27)
**Place of Residence**						
Urban	1.00	reference	1.00	reference	1.00	reference
Rural	1.06	(0.81, 1.38)	1.03	(0.70, 1.53)	1.08	(0.74, 1.56)
**Type of Screening Program**						
Decentralized	1.00	reference				
Centralized	2.75	(1.95, 3.87)				

[a] Estimates for each factor adjusted for the other factors shown and screening site

© 2021 Sakoda LC et al. *JAMA Network Open.*
